# Hsa_circ_0107593 Suppresses the Progression of Cervical Cancer *via* Sponging hsa-miR-20a-5p/93-5p/106b-5p

**DOI:** 10.3389/fonc.2020.590627

**Published:** 2021-01-15

**Authors:** Wenyan Liao, Jun He, Cyrollah Disoma, Yi Hu, Junhua Li, Guodong Chen, Ying Sheng, Xinyu Cai, Chuanfu Li, Kai Cheng, Chunfen Yang, Yongxi Qin, Dong Han, Wu Wen, Chengming Ding, Mujun Li

**Affiliations:** ^1^ Reproductive Medical Center, The First Affiliated Hospital of Guangxi Medical University, Nanning, China; ^2^ Department of Gynaecology and Obstetrics, The First Affiliated Hospital of University of South China, Hengyang, China; ^3^ Department of Hepatopancreatobiliary Surgery, The First Affiliated Hospital of University of South China, Hengyang, China; ^4^ Department of Cell Biology, School of Life Sciences, Central South University, Changsha, China; ^5^ College of Animal Science and Technology, Hunan Agricultural University, Changsha, China

**Keywords:** cervical cancer, circRNA, miRNA, hsa_circ_0107593, hsa-miR-20a-5p, hsa-miR-93-5p, hsa-miR-106b-5p

## Abstract

Circular RNAs (circRNAs) are a new class of single-stranded RNAs that form a continuous loop with crucial role in regulation of gene expression. Because their circular conformation conforms numerous properties, circRNAs have been investigated recently to demonstrate their important role in the development and progression of various cancers. However, the function of circRNAs and their regulatory outcomes in cervical cancer (CC) have rarely been explored. In this study, the role and molecular mechanism of hsa_circ_0107593 in cervical cancer are demonstrated. Quantitative polymerase chain reaction (qRT-PCR) was used to determine the expression of hsa_circ_0107593 and three miRNAs (hsa-miR-20a-5p, 93-5p, and 106b-5p) in paired CC tissues (tumor tissue *vs.* adjacent normal cervical tissue), CC cell lines, and human normal cervical epithelial immortalized cell line. A series of functional experiments were conducted to assess the function of hsa_circ_0107593 in CC development. The Receiver Operating Characteristic (ROC) curve was plotted to estimate the diagnostic value of hsa_circ_0107593 in CC. The dual-luciferase reporter assay was used to explore the interaction between hsa_circ_0107593 and hsa-miR-20a-5p/93-5p/106b-5p. Bioinformatic analysis was conducted to predict the target mRNAs, pathways, and functional enrichment. The results revealed that hsa_circ_0107593 has low expression in CC tissues and CC cell lines. Moreover, negative correlations of hsa_circ_0107593 expression were found against tumor diameter, FIGO stage, and myometrial invasion. Also, hsa_circ_0107593 impedes CC cell proliferation, migration, and invasion. Based on ROC curve analysis, hsa_circ_0107593 could serve as a diagnostic biomarker. Its low expression may indicate increased patient’s risk to developing cervical cancer. Mechanistically, hsa_circ_0107593 serves as a sponge of hsa-miR-20a-5p, hsa-miR-93-5p, and hsa-miR-106b-5p. Collectively, our study implies that hsa_circ_0107593 has tumor-suppressing activity in CC by physically binding with hsa-miR-20a-5p, hsa-miR-93-5p, and hsa-miR-106b-5p.

## Introduction

Cervical cancer (CC) is one of most common gynecological cancers worldwide affecting women (1). In recent years, the morbidity and mortality rates of cervical cancer have gradually increased each year in China (2). Despite obvious improvements in diagnosis and therapy, CC patients’ survival remains unsatisfactory, with a 5-year survival rate of less than 40% (3). Thus, it is necessary to find ideal biomarkers and therapeutic targets to improve CC diagnosis and treatment.

With the emergence of microRNA and long non-coding RNA in the recent years, circRNAs have become a hotspot in disease research (4). CircRNAs are a novel type of endogenous non-coding RNAs, which have attracted a high level of attention due to their critical role in the regulation of gene expression (5). Compared to linear RNAs, circRNAs are more conservative and stable whose functions are specific to certain cell and tissue type as well as to particular developmental stage (6). It has been demonstrated that the possible functions of circRNAs include transcriptional regulation and miRNA sponging (7, 8). Under certain circumstances, circRNAs can be translated directly into proteins (9). Recently, circRNAs have been reported to mediate progression of many cancers (10). For instance, circ_SLC8A1 is downregulated in bladder cancer tissues and cell lines while circ_PIP5K1A is highly expressed in non-small cell lung cancer (NSCLC) cells (11, 12). CircRNAs can also play a role in metastasis. Such is demonstrated by circRNA cRAPGEF5 that inhibits the growth and metastasis of renal cell carcinoma (13). Although circRNAs are now receiving attention, little is known about their role in cervical cancer.

Previously, Liu et al. used Human circRNA Microarray V2.0 to screen CC tissues and three pair-matched adjacent nontumorous tissues. This global profiling in CC yielded 591 differentially expressed circRNAs between CC tissues and pair-matched adjacent nontumorous tissues (14). Hsa_circ_0107593 was among the downregulated circRNAs that are predicted to have crucial regulatory function in cervical carcinogenesis. Hsa_circ_0107593, with designated gene name *ABCA5*, is located in chr17:67270083-67280213 (http://www.circbase.org/).

Nonetheless, the relationship of hsa_circ_0107593 with cervical cancer has not been experimentally validated. In this paper, we hereby presented the association between hsa_circ_0107593 expression and its clinical significance in CC patients. We further investigated the underlying molecular mechanism of hsa_circ_0107593 in CC development and progression.

## Material and Methods

### Collection of Cervical Cancer Tissue Samples

The study was approved by the Medical Ethics Committee of The First Affiliated Hospital of University of South China. All subjects provided written informed consent in accordance with the Declaration of Helsinki principles. Tissue samples were collected from 52 patients who underwent surgery at The First Affiliated Hospital of University of South China from October 2018 to October 2019. None of them received preoperative chemotherapy or radiotherapy. The tissue specimens were immediately preserved in liquid nitrogen after removal from the body and then stored at −80°C until use. Pair-matched normal adjacent tissues were taken 2 cm from the edge of the visible cancerous tumor with no obvious tumor cells. Samples were also sent to three independent pathologists for histopathological analysis for confirmation. The clinicopathological data of all the patients including age, lymph node metastasis, pathologic type, tumor differentiation, tumor diameter, FIGO stage, HPV infection, and myometrial invasion were also collected.

### Cell Culture

The human cervical cancer cell lines (HeLa, SiHa, C-33A, CaSki, C4-1, ME-180) and HEK-293T were gifted by Doctor Fengbo Tan in Xiangya Hospital of Central South University (Changsha, Hunan, China). The human normal cervical epithelial immortalized cell line H8 was purchased from BeNa Culture Collection (BNCC, Kunshan, China). HeLa, SiHa, CaSki, C4-1, and ME-180 cells were cultured in RPMI 1640 medium (Gibco, Thermo Fisher Scientific, Waltham, MA, USA) supplemented with 10% fetal bovine serum (FBS, Gibco). H8 cells were cultured in MEM (Gibco) medium containing 10% FBS, while C-33A and HEK-293T cells were cultured in DMEM (Gibco) medium containing 10% FBS. Then 100 units/ml penicillin and 100 μg/ml streptomycin were added to the culture medium. Cells were cultured in a humidified atmosphere containing 5% CO_2_.

### RNA Extraction and Reverse Transcription

Total RNA was extracted from human tissues and cultured cells by Trizol reagent. Two µg RNA was utilized to reverse transcribed into single-stranded cDNA. Reverse transcription of circRNA was performed using Reverted First Strand cDNA Synthesis Kit (Thermo Fisher Scientific, Vilnius Lithuania) according to the manufacturer’s instructions. The reverse transcription reaction of miRNA was carried out with TaqMan MicroRNA Reverse Transcription Kit (Thermo Fisher Scientific). The purity and concentration of RNA samples were spectrophotometrically quantified by absorbance measurements at 260 and 280 nm using Nanodrop Spectrophotometer.

### Quantitative Real-Time Polymerase Chain Reaction

qRT-PCR was done with the MonAmp™ ChemoHS qPCR Mix (Wuhan, China) in StepOnePlus™ Real-Time PCR System (4376600, Thermo Fisher Scientific, USA). The primers listed in **Table 1** were designed by Primer Premier 5.0 software (Premier, Canada). GAPDH was regarded as the endogenous control for circRNA, while U6 was used as an internal control for normalizing miRNA expression. The primers of circRNA were synthesized by TSINGKE Biotech Ltd. (Beijing, China), and the primers of miRNA were synthesized by Shanghai Sangon Biological Engineering Technology & Services Co., Ltd. (Shanghai, China). All experiments were conducted in triplicate. Fold changes in expression of RNAs were recorded using the 2^−ΔΔCt^ method.

**Table 1:** Primers used for qRT-PCR.

Name	Forward	Reverse
hsa_circ_0107593	AATGCTGTGGTTCCCATC	TCCAGTGGCTGCTGAGTAA
GAPDH	GCACCGTCAAGGCTGAGAAC	TGGTGAAGACGCCAGTGGA
hsa-miR-20a-5p	UAAAGUGCUUAUAGUGCAGGUAG	CUACCUGCACUAUAAGCACUUUA
hsa-miR-106b-5p	UAAAGUGCUGACAGUGCAGAU	GCGAAGAGGTGACAGTGCAGAT
hsa-miR-93-5p	GCCATGTAAACATCTCGGACTG	CAATGCGTGTGGTGGAGGAG
U6	CTCGCTTCG GCAGCACA	AACGCTTCACGAATT TGCGT

### Cell Transfection

For *in vitro* overexpression studies, HeLa and SiHa cells were selected for they showed the lowest expression of hsa_circ_0107593. For knockdown experiments, CaSki and ME180 were selected for they showed the highest expression of hsa_circ_0107593. A plasmid overexpressing hsa_circ_0107593 was transfected into HeLa and SiHa cells. siRNAs targeting hsa_circ_0107593 were transfected into CaSki and ME180 cells. Three siRNAs (siRNA-1, siRNA-2, and siRNA-3) and corresponding negative control siRNA (si-NC) were purchased from RiboBio (Guangzhou, China). The siRNA with highest knockdown efficiency was selected for all subsequent experiments. The overexpressing plasmid and its corresponding negative control plasmid (OE-NC) were purchased from TSINGKE Biotech Ltd. (Beijing, China). Cells were harvested 48 h post-transfection, then transfection efficiency was checked by qRT-PCR. For dual-luciferase reporter assay, hsa-miR-20a/93/106b-5p mimics, as well as their respective negative control (NC mimics) were purchased from Shanghai Sangon Biological Engineering Technology & Services Co., Ltd. Cell transfection was performed with Lipofectamine 3000 (Invitrogen, Thermo Fisher Scientific, USA) following the manufacturer’s instructions.

### Cell Proliferation Assay

After 48 h post-transfection with overexpressing plasmids and siRNAs, the cell proliferation assay was performed using Cell Counting Kit-8 (CCK-8, Vazyme Biotech, Nanjing, China). Briefly, a total of 100 µl cells were plated into 96-well plates at a density of 2 × 10^3^ each well with serum-free RPMI 1640 medium. It was replicated with three independent wells. Cell proliferation was measured at 24, 48, and 72 h after seeding cells. Before observation, each well was treated with 10 μl CCK-8 reagent and then cells were incubated at 37℃ for another 3 h before measuring absorbance at 450 nm using a microplate reader (Bio-Rad, Hercules, CA, USA). All experiments were repeated thrice.

### Colony Formation Assay

Colony-forming ability was evaluated using a six-well tissue culture plates. Transfected cells were resuspended with culture medium and inoculated on a six-well plate with a density of 500 cells/well. The cells were cultured at 5% CO_2_ at 37°C for 12 days. The cells were washed by phosphate buffer saline (PBS) three times, then fixed with methanol for 30 min and stained with 0.1% crystal violet. Colonies were counted using a light microscope.

### *In Vitro* Wound-Healing Assay

Transfected cells were seeded onto 24-well plates and were cultured until 90% confluency. After serum starvation for 6 h, an artificial wound was made in the cell monolayer with a sterile 10 μl pipette tip. After incubation for 24 h, the percentage of wound closure was determined from three independent experiments.

### Transwell Migration Assay and Invasion Assay

Transfected cells were resuspended in 250 μl serum-free medium and seeded into the upper chamber of 24-well plates of the transwell system (Corning, New York, NY, USA) with or without Matrigel-coated membranes (BD Biosciences, New York, NY, USA) for cell invasion and migration assay. For migration assay, 2.5 × 10^4^ cells were placed into chambers of transwell inserts without basement membrane coating. For the invasion assay, 5 × 10^4^ cells were seeded in chambers of transwell inserts with a basement membrane coating. While 450 μl medium containing 20% FBS were supplied at the lower chamber as chemoattractant. After incubation at 5% CO_2_/37°C for 24 h, residual cells on the upper surface of the membrane were removed with a cotton swab, while cells that had traversed through the membrane were fixed with methanol for 30 min and then stained with 0.1% crystal violet for 30 min. Cells were counted using a microscope and the relative migration rate or invasion rate were calculated.

### Dual-Luciferase Reporter Assay

We used an online bioinformatic program miRanda to predict the binding sites to construct the recombinant luciferase vectors carrying wild type (WT) and mutant (Mut) hsa_circ_0107593. The WT and Mut sequences were synthesized by TSINGKE Biotech Ltd. Then WT and Mut hsa_circ_0107593 were inserted into pMIR-REPORT vector (Ambion, Waltham, MA, USA). HEK-293T cells were cotransfected with hsa-miR-20a/93/106b-5p mimics or NC-mimics, and the recombinant luciferase vectors using Lipofectamine 3000. After a 48 h incubation, luciferase activity was detected using a dual-luciferase reporter assay system (Promega Corporation) according to the manufacturer’s protocol. Renilla luciferase activity was detected and used as the internal control.

### Bioinformatic Analysis

The target miRNAs of hsa_circ_0107593 were predicted by online software miRanda (http://www.miranda.org/) and RNAplex (http://www.bioinf.uni-leipzig.de/~htafer/RNAplex/RNAplex.html), and target mRNAs for those miRNAs were predicted by the online software miRanda. Network map was drawn using Cytoscape Software (http://www.cytoscape.org). Gene ontology (GO) enrichment analysis and KEGG pathway analysis were carried out based on goatool (https://github.com/tanghaibao/goatools) and KOBAS (http://kobas.cbi.pku.edu.cn/kobas3/?t=1). While GO and KEGG analysis were performed for targeted mRNAs. The complete datasets were uploaded to www.mendeley.com (https://dx.doi.org/10.17632/s78nsw6v32.1).

### Statistical Analysis

All quantitative data were presented as mean ± standard deviation (SD) as indicated from at least three independent experiments. Statistical analyses were performed using GraphPad Prism 7.0 Software for Windows (GraphPad Software, La Jolla, CA, USA) and Service Solutions SPSS Software 19.0 (SPSS, Chicago, IL, USA). Between-group differences were tested for significance using one-way analysis of variance and Student’s t-test. The associations between circRNA expression and the clinicopathological parameters of CC were assessed by the Chi-square test. The receiver operating characteristic (ROC) curve was plotted to evaluate diagnostic values. Correlation analysis was performed using Spearman’s rank correlation coefficient. P < 0.05 was considered statistically significant.

## Results

### Hsa_circ_0107593 Is Downregulated in Cervical Cancer

**Figure 1:**
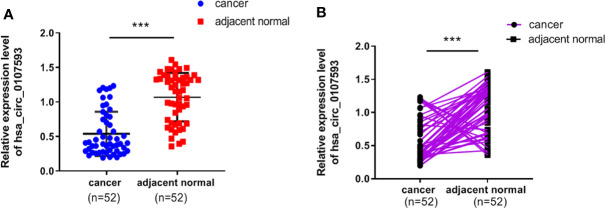
Low expression level of hsa_circ_0107593 in CC tissues. **(A)** The overall expression level of hsa_circ_0107593 was downregulated in CC tissues by qRT-PCR. **(B)** Hsa_circ_0107593 expression was assayed in 52 pairs of CC tissues and matched normal tissues by qRT-PCR. (***P-value <0.001).

To explore the differential expression of hsa_circ_0107593 in cervical cancer, we performed qRT-PCR analysis on 52 patient specimens. The overall expression level of hsa_circ_0107593 was significantly downregulated in CC tissues compared with their adjacent normal tissues. (p < 0.001, **Figure 1A**). When paired T test was used, the expression of hsa_circ_0107593 was also significantly downregulated in CC tissues compared with their matched adjacent normal tissues (p < 0.001, **Figure 1B**). When bounded by p < 0.05, significant downregulation in tumor tissue was observed in 45 paired tissues. In the remaining 7 paired tissues, no significant differences were noted between CC tissues compared with their matched adjacent normal tissues. Together, the expression of hsa_circ_0107593 was significantly downregulated in 86.54% (45/52) CC tissues compared with their adjacent normal tissues. The downregulation of hsa_circ_0107593 is consistent in six human CC cell lines (HeLa, SiHa, C-33A, CaSki, C4-1, and ME-180). In contrast, hsa_circ_0107593 is more expressed in the immortalized normal cervical cell line H8. This indicates that hsa_circ_0107593 could be related to cervical cancer.

### Expression of hsa_circ_0107593 and Clinicopathological Characteristics of Cervical Cancer Patients

To further demonstrate the clinical significance of hsa_circ_0107593 downregulation, we performed correlational analyses of hsa_circ_0107593 expression with clinicopathological characteristics of CC patients. Our patient cohort was divided into two groups using the median expression level as cutoff value: high-expression group and low-expression group. The level of hsa_circ_0107593 expression was significantly correlated with tumor diameter (P = 0.009), FIGO (International Federation of Gynecology and Obstetrics) stage (P = 0.023), and myometrial invasion (P = 0.025). On the other hand, there were no significant associations between hsa_circ_0107593 expression and the other clinicopathological parameters including age (p = 0.577), lymph node metastasis (p = 0.465), pathologic type (p = 0.324), HPV infection (p = 0.174), and tumor differentiation (p = 0.075) (**Table 2**).

**Table 2:** Correlation between hsa_circ_0107593 expression levels (2^−ΔΔCt^) and clinicopathological characteristics of cervical cancer patients.

Clinicopathological Characteristics		Number of Patientsn = 52	Expression of hsa_circ_0107593		P value
			Lower (n = 26)	Higher (n = 26)	
Age (years)	<45	23	10	13	0.577
	≥45	29	16	13	
FIGO stage	Ib	31	11	20	0.023*
	IIa-IIb	21	15	6	
Lymph node metastasis	Negative	43	20	23	0.465
	Positive	9	6	3	
myometrial invasion	<1/2	29	10	19	0.025*
	≥1/2	23	16	7	
Tumor diameter (cm)	<4	39	15	24	0.009*
	≥4	13	11	2	
Pathological type	Squamous cell carcinoma	40	18	22	0.324
	Adenocarcinoma	12	8	4	
Tumor differentiation	Well and mediate	42	18	24	0.075
	Poor	10	8	2	
HPV infection	Negative	11	3	8	0.174
	Positive	41	23	18

*Significant association (*P-value < 0.05).

### Overexpression of hsa_circ_0107593 Impedes HeLa Cell Proliferation, Migration, and Invasion

**Figure 2:**
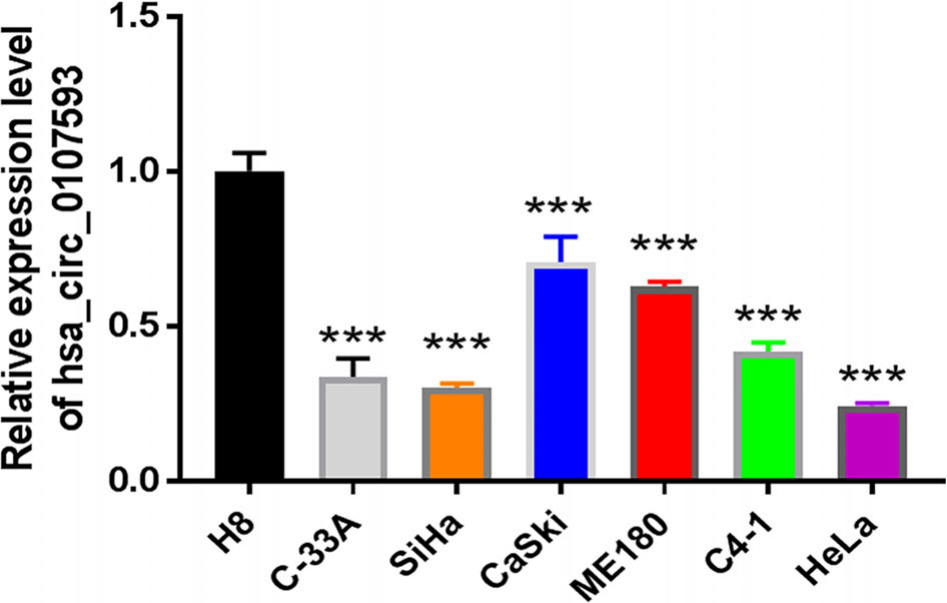
The expression of hsa_circ_0107593 was much lower in CC cell lines than human normal cervical epithelial immortalized cell line H8 by qRT-PCR. (***P-value <0.001).

**Figure 3:**
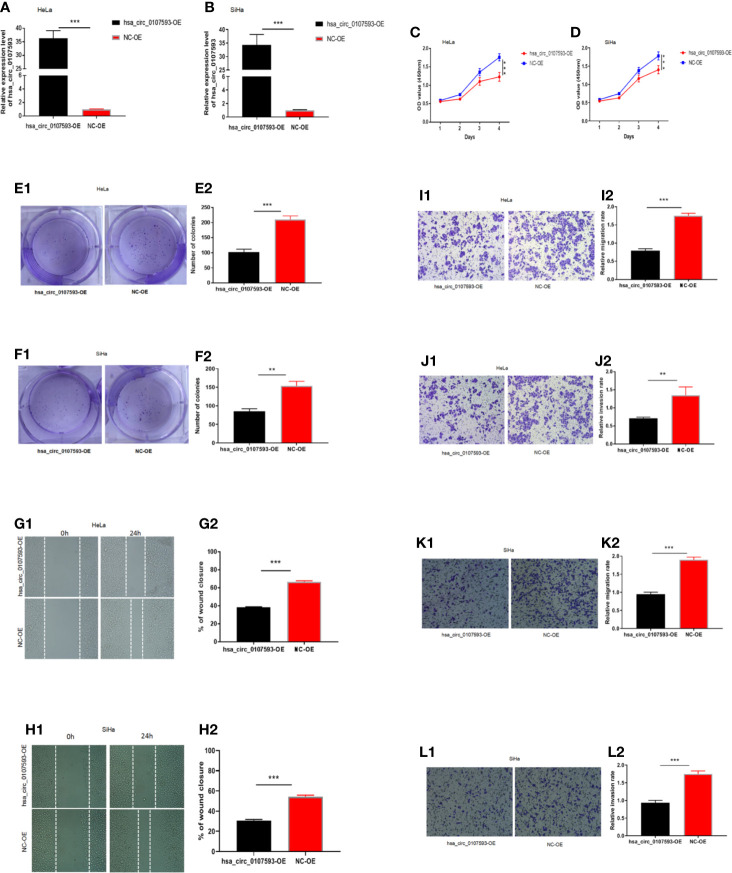
Overexpression of hsa_circ_0107593 impedes CC cells proliferation, migration, and invasion. **(A, B)** Overexpression of hsa_circ_0107593 was validated by qRT-PCR in CC cells. **(C, D)** The CCK-8 assay showed that the proliferation rate of CC cells in hsa_circ_0107593 overexpressed group was significantly reduced than control group. **(E, F)** Effects of hsa_circ_0107593 overexpression on the colony-forming capacity of CC cells was assessed using a colony formation assay. The results showed that overexpression of hsa_circ_0107593 inhibited the colony-forming capacity of CC cells. **(G, H)** Cell migration was determined by *in vitro* wound-healing assay. The results showed that overexpression of hsa_circ_0107593 impeded CC cell migration. **(I–L)** Cell migration and invasion were determined by transwell assays. The transwell migration and invasion assay showed that overexpression of hsa_circ_0107593 inhibited the migration and invasion of CC cell. (***P-value < 0.001, **P-value < 0.01). hsa_circ_0107593-OE, hsa_circ_0107593 overexpressed group; NC-OE, negative control.

In our screening of several CC cell lines, we found out that HeLa and SiHa cells had the lowest hsa_circ_0107593 expression (**Figure 2**). For this reason, HeLa and SiHa were the best two cell lines to model cervical cancer for further *in vitro* functional analyses. The transient overexpression of hsa_circ_0107593 significantly decreased cell proliferation (**Figures 3A–D**). Additionally, the colony formation assay demonstrated that overexpression of hsa_circ_0107593 significantly repressed the colony-forming capability of HeLa cells (**Figures 3E, F**). *In vitro* wound-healing assay further indicated that overexpressing hsa_circ_0107593 significantly impairs the migration capacity of CC cells (**Figures 3G, H**). Moreover, transwell migration assay and invasion assay confirmed that the migration rate and invasion rate of hsa_circ_0107593-overexpressed CC cells were significantly lower than those of the control (**Figures 3I–L**). Altogether, these data demonstrated an anti-cancer role of hsa_circ_0107593 in CC cells.

### Knockdown of hsa_circ_0107593 Promotes CaSki Cell Proliferation, Migration, and Invasion

**Figure 4:**
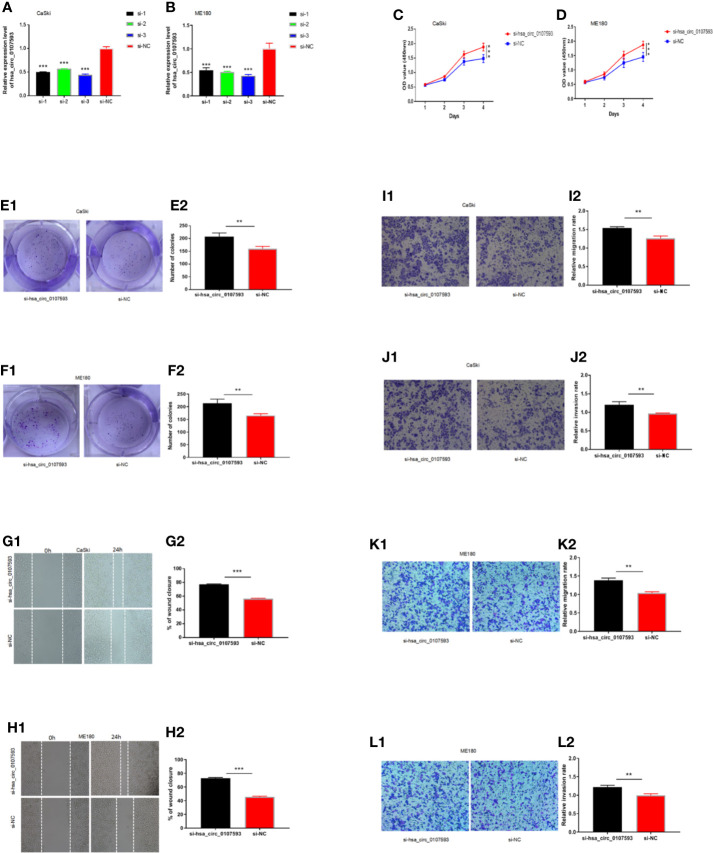
Knockdown of hsa_circ_0107593 promotes CC cell proliferation, migration, and invasion. **(A, B)** The expression levels of hsa_circ_0107593 in CC cells were determined by qRT‐PCR after transfection with three hsa_circ_0107593 siRNAs individually (siRNA-1, siRNA-2, and siRNA-3). The results showed that siRNA-3 is the most effective siRNA, so we selected siRNA-3 for all subsequent experiments. **(C, D)** The CCK-8 assay showed that the proliferation rate of CC cells in si-hsa_circ_0107593 group was significantly increased than control group. **(E, F)** Effects of hsa_circ_0107593 knockdown on the colony-forming capacity of CC cells was assessed using a colony formation assay. The results showed that knockdown of hsa_circ_0107593 significantly increased the colony-forming capability of CC cells. **(G, H)** Cell migration was determined by *in vitro* wound-healing assay. The results showed that knockdown of hsa_circ_0107593 promoted CC cell migration. **(I–L)** Cell migration and invasion were determined by transwell assays. The transwell migration and invasion assay showed that knockdown of hsa_circ_0107593 could facilitate the migration and invasion of CC cell. (***P-value < 0.001, **P-value < 0.01). si-hsa_circ_0107593, hsa_circ_0107593 knockdown group; si-NC, negative control.

Meanwhile, CaSki and ME180 cells had the highest expression of hsa_circ_0107593. We selected these two cell lines to investigate the loss of function of hsa_circ_0107593 *in vitro*. The knockdown of hsa_circ_0107593 using three siRNAs were validated by qRT-PCR. Of these three, siRNA-3 showed the best interference efficiency in CaSki and ME180 cells (**Figures 4A, B**). Therefore, siRNA-3 was selected in the succeeding experiments. The knockdown of hsa_circ_0107593 significantly promoted cell proliferation (**Figures 4C, D**), increased the colony-forming capability (**Figures 4E, F**), and facilitated the migration capacity of CC cells (**Figures 4G, H**). Moreover, transwell migration assay and invasion assay confirmed that the migration rate and invasion rate of knocking down hsa_circ_0107593 in CC cells were significantly higher than those of the control cells (**Figures 4I–L**). These results clearly demonstrated that hsa_circ_0107593 plays a tumor-suppressing role in cervical cancer. Its low expression can promote development of cervical cancer and potentially increases its metastatic potential.

### Diagnostic Value of hsa_circ_0107593 in Cervical Cancer

**Figure 5:**
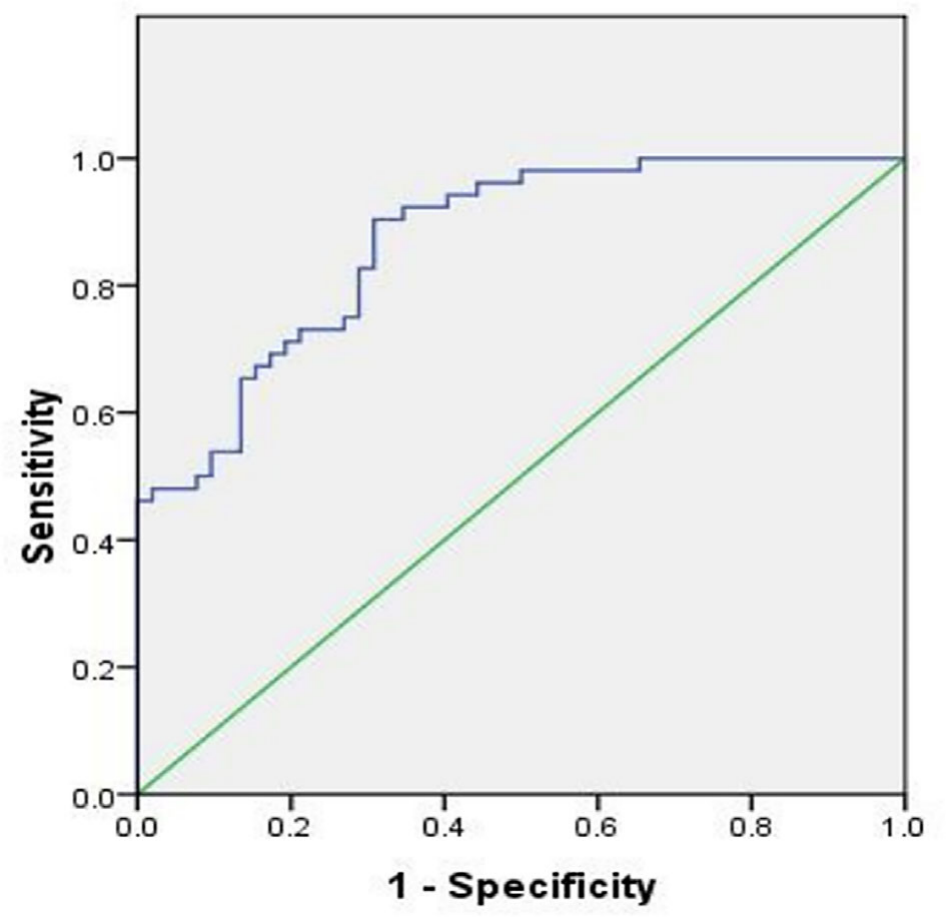
ROC curve was conducted to evaluate the potential diagnostic value of hsa_circ_0107593 in CC (AUC = 0.869, 95% CI = 0.803–0.934, P < 0.001).

The Receiver Operating Characteristic (ROC) curve was plotted using hsa_circ_0107593 expression level in CC tissues against its pair-matched adjacent normal tissues to estimate the diagnostic value of hsa_circ_0107593. The area under the ROC curve (AUC) was 0.869, suggesting that hsa_circ_0107593 might serve as a diagnostic biomarker for CC (**Figure 5**). Upon using the cutoff value of 0.584, the Youden index, sensitivity, and specificity were 0.596, 0.904, and 0.692, respectively.

### Hsa_circ_0107593 Interacts With hsa-miR-20a-5p, hsa-miR-93-5p, and hsa-miR-106b-5p

**Figure 6:**
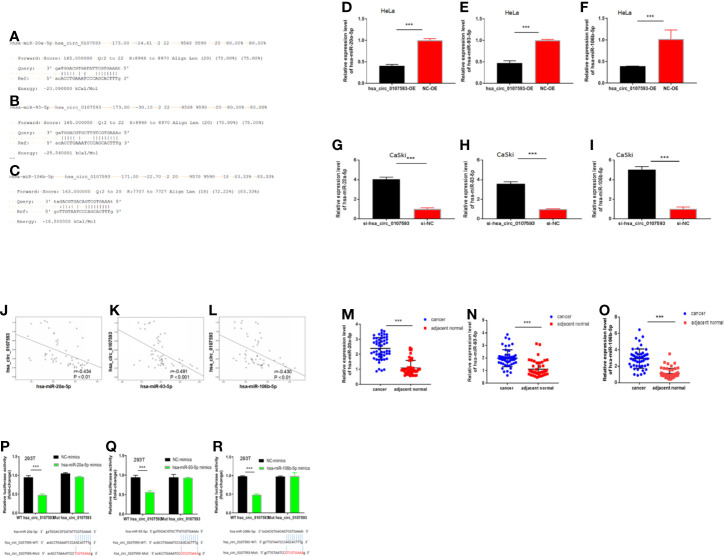
Hsa_circ_0107593 interacts with hsa-miR-20a-5p/93-5p/106b-5p. **(A–C)** Predicted binding site of hsa-miR-20a-5p/93-5p/106b-5p in hsa_circ_0107593 by bioinformatics analysis. **(D–F)** qRT-PCR analysis showed that overexpression of hsa_circ_0107593 down-regulated hsa-miR-20a-5p/93-5p/106b-5p expression in HeLa cells. **(G–I)** qRT-PCR analysis showed that knockdown of hsa_circ_0107593 promoted hsa-miR-20a-5p/93-5p/106b-5p expression in CaSki cells. **(J–L)** Expression of hsa-miR-20a-5p/93-5p/106b-5p and hsa_circ_0107593 were measured by qRT-PCR in CC tissues, then the correlation between hsa-miR-20a-5p/93-5p/106b-5p and hsa_circ_0107593 was analyzed by Spearman’s rank correlation coefficient. **(M–O)** qRT-PCR analysis showed that hsa-miR-20a-5p/93-5p/106b-5p was significantly upregulated in CC tissues. **(P–R)** Dual-luciferase reporter assays in HEK-293T cells showed that hsa-miR-20a-5p/93-5p/106b-5p significantly decreases the relative luciferase activity of the wild-type hsa_circ_0107593 luciferase plasmid compared with the mutant. (***P-value < 0.001).

Because our tissue-based and cell-based analyses provided clues that the low expression of hsa_circ_0107593 promotes tumor formation, we performed further experiments to elucidate how hsa_circ_0107593 perform this role. Using the online bioinformatic tools miRanda and RNAplex, three microRNAs were predicted to be potential targets of hsa_circ_0107593: hsa-miR-20a-5p, hsa-miR-93-5p, hsa-miR-106b-5p. The bioinformatics prediction also showed that hsa_circ_0107593 could harbor those three miRNAs by miRNA seed sequence matching (**Figures 6A–C**). To validate these predictions, we overexpressed hsa_circ_0107593 in HeLa cells and then performed qRT-PCR to detect the levels of these three miRNAs. The overexpression of hsa_circ_0107593 significantly reduced the expression of the predicted targets hsa-miR-20a-5p, hsa-miR-93-5p, and hsa-miR-106b-5p (**Figures 6D–F**). This is consistent with the knockdown experiments demonstrating that the loss of hsa_circ_0107593 in CaSki cells upregulated the expression of the three target miRNAs (**Figures 6G–I**). Further, there was a strong negative correlation between hsa_circ_0107593 and hsa-miR-20a-5p/93-5p/106b-5p expression in CC tissues (**Figures 6J–L**). Not surprisingly, hsa-miR-20a-5p, 93-5p, and 106b-5p expressions were upregulated in CC tissues (**Figures 6M–O**). To further verify the interaction of hsa_circ_0107593 with these miRNAs, we then performed a luciferase reporter assay for the three miRNAs. The results showed that overexpression of hsa-miR-20a-5p/93-5p/106b-5p in HEK-293T cells significantly inhibited the luciferase activity of hsa_circ_0107593-WT reporter plasmid whereas the luciferase activity of hsa_circ_0107593-Mut constructs was not affected (**Figures 6P–R**). These results indicated that hsa_circ_0107593 inhibits hsa-miR-20a-5p/93-5p/106b-5p expression by directly sponging hsa-miR-20a-5p/93-5p/106b-5p.

### Hsa_circ_0107593 Contributes to Cervical Cancer Cell Proliferation, Migration, and Invasion *via* Regulation of hsa-miR-20a-5p/93-5p/106b-5p

**Figure 7:**
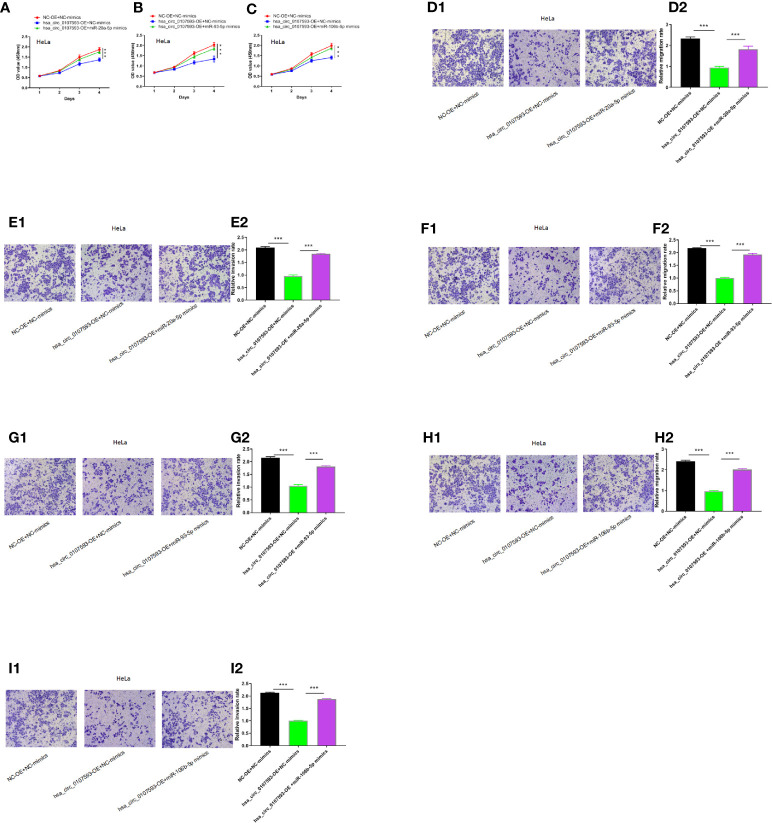
Hsa_circ_0107593 contributes to CC cell proliferation, migration, and invasion *via* regulation of hsa-miR-20a-5p/93-5p/106b-5p. **(A–C)** Cell proliferation was monitored by CCK-8 assay. **(D–I)** Cell migration and invasion was detected by transwell migration and invasion assay. The results showed that upregulation of hsa-miR-20a-5p/93-5p/106b-5p could significantly rescue the suppression effects of hsa_circ_0107593. (***P-value < 0.001).

To determine whether the hsa_circ_0107593 could affect cell proliferation, migration and invasion in CC *via* sponging hsa-miR-20a-5p/93-5p/106b-5p, we performed CCK8 assays using HeLa cells transfected with hsa_circ_0107593 overexpressing plasmid/corresponding negative control plasmid, together with the hsa-miR-20a-5p/93-5p/106b-5p mimics or NC-mimics. As shown in **Figures 7A–I** the results demonstrated that hsa_circ_0107593 overexpression remarkably suppressed CC cell proliferation, whereas up-regulation of hsa-miR-20a-5p/93-5p/106b-5p in the meantime could significantly rescue the suppression effects of hsa_circ_0107593. Overall, these results demonstrated that hsa_circ_0107593 acts as a tumor suppressant by regulating the activity of hsa-miR-20a-5p/93-5p/106b-5p.

### Prediction of circRNA–miRNA–mRNA Interaction, Gene Ontology, and KEGG Pathway Annotation for Targeted mRNAs

**Figure 8:**
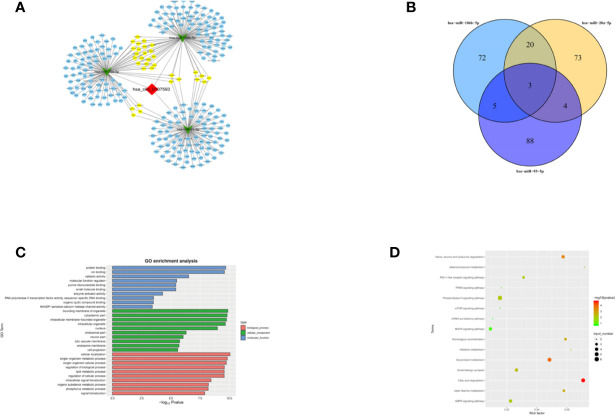
Prediction of the circRNA-miRNA-mRNA interaction, GO and KEGG pathway annotation for targeted mRNAS. **(A)** A network map comprising hsa_circ_0107593, three miRNAs, and their downstream targets was presented. **(B)** Venn diagram revealed the number of common downstream targets of three miRNAs. **(C)** The hsa_circ_0107593-miRNA- mRNA related GO analysis. **(D)** The hsa_circ_0107593-miRNA-mRNA related KEGG pathway analysis.

To further explore the molecular mechanism of hsa_circ_0107593, we investigated the potential mRNAs bound to hsa-miR-20a-5p/93-5p/106b-5p. The downstream targets of the three miRNA were presented in **Figure 8A** generated using Cytoscape software. The three miRNAs have overlapping downstream targets, suggesting that these microRNAs play similar roles (**Figure 8B**). The target genes were involved in various biological functions and signaling pathways as revealed by GO and KEGG pathway analyses (**Figures 8C, D**). The hsa_circ_0107593-miRNA-mRNA related GO analyses fell into three main categories: cellular component, molecular function, and biological process. In the cellular component domain, bounding membrane of organelle were the most represented terms. Protein binding was the most represented GO term under the molecular function domain. Lastly, many target mRNAs are involved in a lot of biological function processes such as cellular localization, intracellular signal transduction, and so on. Interestingly, the target mRNAs were also closely related with various cancer-related pathways, such as fatty acid degradation, AMPK, and phospholipase D signaling pathway.

## Discussion

CircRNAs were first discovered in RNA viruses in the 1970s. Because of technological and methodological limitations at that time, the detected circRNAs were generally regarded as rare splicing “mistakes” and did not receive much attention until recently (15). Since sequencing technology coupled with bioinformatics approaches has improved dramatically in the recent years, many circRNAs have been predicted and are now being explored to identify their role in physiological and pathological states. CircRNAs have recently emerged as a new class of endogenous RNAs that are formed by a covalently closed continuous loop without 5′ caps and 3′ tails compared to mRNAs, and exist extensively in mammalian cells (16, 17). CircRNAs are structurally stable, tissue-specific, and evolutionarily conserved, and usually exhibit tissue- or developmental-stage-specific expression. In addition, they cannot be degraded by RNase. Many circRNAs are derived from the exon sequence of genes that encode proteins. Only a few circRNAs are derived from introns. A large number of circRNAs do not perform protein-coding functions in cells and are therefore considered non-coding RNAs (18, 19). Previous studies have shown that the accumulation of circRNAs is involved in translation regulation through controlling modification and gene expressions (20).

In recent years, an increasing attention is poured into circRNAs in search of their clinical significance in cancer (21–23). Their abnormal expression has been detected in many malignant tumors, suggesting that circRNAs may play an important role in the occurrence and development of malignant tumors. This new area of research is extra attractive for their applications in clinical practice as tumor marker or molecular therapeutic targets are possible (24–26). In a few available studies, circRNAs are reported to play the role of oncogene or tumor suppressor gene, affecting the occurrence of tumors development, such as lung squamous cell carcinoma, colorectal cancer, hepatocellular carcinoma, thyroid cancer, and so on (27–30). However, there have been little studies on circRNAs in gynecologic malignancies.

In this study, we showed that hsa_circ_0107593 was downregulated in CC tissue and CC cell lines, illustrating that low expression of hsa_circ_0107593 might prompt progression of CC. We found that overexpression of hsa_circ_0107593 in CC cells impeded CC cells proliferation, migration, and invasion, while knockdown of hsa_circ_0107593 in CC cells promoted cell proliferation, migration, and invasion, suggesting that hsa_circ_0107593 possesses potent anti-cancer abilities in CC. Our study implies that increasing hsa_circ_0107593 expression might be an effective gene therapy for inhibiting tumor development, especially for CC patients with low expression in their cancer tissue. Moreover, we found that hsa_circ_0107593 negatively correlated with tumor size, FIGO stage, and myometrial invasion. This is consistent with our findings that hsa_circ_0107593 can inhibit the proliferation, invasion, and migration of cervical cancer cells *in vitro*. But hsa_circ_0107593 is not related to the other clinicopathological parameters such as age, lymph node metastasis, pathologic type, HPV infection, and tumor differentiation. In addition, we constructed a ROC curve for differentiating CC tissues from pair-matched adjacent normal tissues to estimate the diagnostic value of hsa_circ_0107593 in CC. The calculated values suggest that hsa_circ_0107593 might serve as a potential biomarker for the diagnosis of CC. Therefore, hsa_circ_0107593 may serve as a new biomarker and a potential therapeutic target for CC. However, it is needed to acquire a more robust data that can support the significance of hsa_circ_0107593 as tumor biomarker and as meaningful clinical tool for diagnosis.

The regulatory function of circRNAs is mainly due to their competitive binding with miRNAs. In turn, this binding affects the function of miRNAs and the subsequent expression levels of their downstream target genes (31). These circRNA-miRNA axes are involved in many disease pathways such as apoptosis, vascularization, invasion, and metastasis during carcinogenesis (32). From our bioinformatics and luciferase reporter assay analysis, we found that hsa_circ_0107593 could interact with three miRNAs: hsa-miR-20a-5p, 93-5p, and 106b-5p. Rescue experiments showed that hsa-miR-20a-5p/93-5p/106b-5p mimics can reverse the tumor-suppressing roles of hsa_circ_0107593 in CC. Further probing using bioinformatics analysis resulted to identification of many targets, which are involved in a lot of biological function processes such as cellular metabolism, protein binding, cellular localization, and so on. The function of these target genes can be altered by hsa_circ_0107593 *via* sponging the three miRNAs. Importantly, hsa_circ_0107593 was closely related with a lot of cancer-related pathways, such as fatty acid degradation, AMPK, and phospholipase D signaling pathway. This means that hsa_circ_0107593 may play a tumor suppressor role by modulating these pathways. However, further studies are needed to uncover the molecular mechanism of hsa_circ_0107593 in these cancer-related pathways in the context of cervical cancer and even in other malignancies.

In conclusion, our study revealed that the expression of hsa_circ_0107593 is decreased in CC tissue and cell lines. Its low expression contributes to the progression of the disease. The ROC curve suggested that hsa_circ_0107593 may be highly valuable in the diagnosis of CC, and that it might be a novel potential diagnostic biomarker. Moreover, hsa_circ_0107593 acts by sponging hsa-miR-20a-5p/93-5p/106b-5p to inhibit the tumor-promoting activities of those miRNAs in CC. This regulatory mechanism of hsa_circ_0107593 inhibits CC cell proliferation, migration, and invasion. The hsa_circ_0107593/hsa-miR-20a-5p/93-5p/106b-5p axis provided a novel insight into the molecular mechanisms of CC. These findings may provide new information for the diagnosis and treatment of CC.

## Data Availability Statement

The raw data supporting the conclusions of this article will be made available by the authors, without undue reservation.

## Ethics Statement

The study was approved by the Medical Ethics Committee of The First Affiliated Hospital of University of South China. All subjects provided written informed consent in accordance with the Declaration of Helsinki principles.

## Author Contributions

WL contributed to the manuscript and main experiments. JH, YH, and GC: analyzed the figures and data. ChD and ML designed the study. CyD, YH, JL, GC, YS, XC, CL, KC, CY, YQ, DH, and WW assisted in the experiment and revised the manuscript. All authors contributed to the article and approved the submitted version.

## Funding

This work was supported by Hunan Provincial Natural Science Fund (2018JJ3471), Scientific Research Fund Project of Hunan Provincial Health Commission (C20180156, 20201950).

## Conflict of Interest

The authors declare that the research was conducted in the absence of any commercial or financial relationships that could be construed as a potential conflict of interest.
